# Are functional outcomes and early pain affected by discharge on the day of surgery following total hip and knee arthroplasty?

**DOI:** 10.1080/17453674.2020.1836322

**Published:** 2020-10-26

**Authors:** Christian E Husted, Henrik Husted, Lina Holm Ingelsrud, Christian Skovgaard Nielsen, Anders Troelsen, Kirill Gromov

**Affiliations:** Department of Orthopedic Surgery, Copenhagen University Hospital, Hvidovre, Denmark

## Abstract

Background and purpose — Outpatient total knee and total hip arthroplasty (TKA and THA) has been shown to be feasible and safe in selected patients. However, little data is available on functional outcome and early pain in patients discharged on the day of surgery (DOS). We investigated patient-reported outcomes at 1 year and early pain in outpatient TKA and THA patients discharged on the day of surgery (DOS) (DDOS) compared with patients scheduled for outpatient surgery but not discharged on the DOS (nDDOS).

Patients and methods — Prospective data on 261 consecutive patients scheduled for outpatient TKA (n = 126) and THA (n = 135) were collected. 37% of TKA patients and 33% of THA patients were discharged on the DOS. Pain scores at rest and activity and use of morphine were registered on postoperative days 1–7. Oxford Knee Score (OKS) and Oxford Hip Score (OHS) were collected preoperatively and at 3 and 12 months’ follow-up.

Results — DDOS and nDDOS patients were similar in respect to age, sex, procedure type (TKA vs. THA), or preoperative OKS or OHS. Neither OKS nor OHS differed between groups at 3 and 12 months’ follow-up. Pain at rest and activity and use of morphine did not differ between the 2 groups on days 1–7.

Interpretation — In patients scheduled for outpatient TKA and THA, we found similar patient-reported outcomes both early and at 1 year in those discharged on the DOS and those who had at least 1 overnight stay.

Fast-track total knee arthroplasty (TKA) and total hip arthroplasty (THA) have gained significant popularity over several years due to the many favorable aspects of optimizing perioperative care in these procedures. These aspects include reduced perioperative mortality and morbidity, reduced length of stay (LOS), and reduced financial expenses (Andreasen et al. 2017, Burn et al. 2018).

Outpatient surgery has become increasingly popular in recent years in selected patients, and all the appealing aspects of standardized outpatient arthroplasty have been reported to come with no increased risk to patients’ safety (Pollock et al. 2016, Goyal et al. 2017, Vehmeijer et al. 2018, Gromov et al. 2019, Xu et al. 2019) and at even lower cost (Husted et al. 2018).

One of the potential concerns regarding outpatient THA and TKA is the rehabilitation that patients receive during hospital stay which can be as short as a few hours, which potentially can affect postoperative pain and functional outcome. While safety aspects following outpatient THA and TKA in selected patients have been thoroughly investigated, little information exists regarding the implications that discharge on the DOS has on postoperative pain and functional outcome.

Therefore, we investigated the degree of pain in the first postoperative week, as well as pain and functional outcomes at 1 year following outpatient TKA and THA among patients discharged on the DOS (DDOS) and compared them with TKA and THA patients scheduled for outpatient surgery but not discharged on the DOS (nDDOS).

## Patients and methods

Patients undergoing primary unilateral THA and TKA between January 2016 and June 2017 at our high-volume center and scheduled for same-day discharge were included in this study. Patient selection and eligibility for a part of this cohort was previously described (Gromov et al. 2017). In brief, patients were considered eligible for inclusion in this study if they did not suffer from sleep apnea and had ASA scores of < 3. Furthermore, only patients who were operated on as 1st or 2nd in the surgical theater were included.

All operations were performed in a standardized fast-track setup (Husted 2012) by surgeons specialized in THA and TKA surgery. The standard surgical protocol for both THA and TKA included intended spinal anesthesia, standardized fluid management, preoperative single-shot high-dose methyl­prednisolone (Lunn et al. 2011, 2013), use of preoperative tranexamic acid (TXA)—THA patients received 2 doses IV, and TKA patients received an additional intra-articular dose—and absence of drains. All THAs were performed using a standard posterolateral approach. All TKAs were performed with a standard medial parapatellar approach without the use of a tourniquet and using local infiltration analgesia (LIA) (Andersen and Kehlet 2014). The patients were transferred from the postoperative recovery unit to the patient ward after a few hours, where mobilization was attempted as soon as possible, allowing full weight-bearing. Rivaroxaban was used as oral thromboprophylaxis, starting 6–8 hours postoperatively and continuing daily until discharge. No extended thromboprophylaxis was used.

Pain medication consisting of celecoxib 200 mg/12 hours and paracetamol 1 g/6 hours were given until postoperative day 7 (POD 7). No opioids were systematically given to any patients and oral morphine 10 mg pro necessitate was used as rescue analgesic only.

Physiotherapy was started on the DOS and continued until discharge. All patients were referred to public outpatient physio­therapy, which they attended as long as seemed fit by the treating therapist.

Patients were discharged if discharge criteria were fulfilled before 8 p.m. on the DOS. These criteria included patients not exceeding 500 mL of intraoperative blood loss and pain scores < 3 (VAS) while resting and < 5 during weight-bearing mobilization. Furthermore, urination had to be spontaneous and mobilization had to be achieved with a physiotherapist. Also, an adult had to be present with the patient for the first 24 hours after discharge. Patients not fulfilling discharge criteria on the DOS stayed overnight until discharge criteria were fulfilled.

Patient-reported outcomes were measured using Oxford Knee Score (OKS) and Oxford Hip Score (OHS)—a 12-item questionnaire regarding knee/hip pain and function that are summed to a total score of 0–48 (worst–best) (Murray et al. 2007). OKS and OHS were registered preoperatively as well as 3 months and 1 year after THA or TKA.

Following surgery, the patients were asked to fill out a questionnaire regarding daily pain at rest as well as during activity. This was measured on the VAS 0–10, 10 being worst imaginable pain, and daily use of morphine (yes/no) was recorded on postoperative days 1–7. The questionnaire was given to the patients upon discharge and collected at 3 months’ follow-up.

Later, overall satisfaction with treatment was recorded using a numeric rating scale (NRS) 0–10, 10 being very satisfied, and willingness to undergo the same treatment again (yes/no/I don’t know) was answered at 3 months’ follow-up.

### Statistics

Normality assumption for all continuous variables was evaluated by Q–Q plots as well as skewness and kurtosis measurements. Mean values (SD) are presented for normally distributed variables, while median values and interquartile ranges (IQR) are presented for non-normally distributed variables. The Mann–Whitney U-test was used to compare continuous non-parametric variables, Student’s t-test to compare continuous parametric variables, and a chi-square test to compare categorical variables.

All data were processed in R 3.2.2 (R Foundation for Statistical Computing, Vienna, Austria).

### Ethics, funding, and potential conflicts of interest

No approval from the National Ethics Committee was necessary as this was a non-interventional observational study. The study was approved by the Danish Data Protection Agency (entry no. 20047-58-0015). This work was sponsored by grants from the Lundbeck Foundation and ZimmerBiomet, which had no influence on any part of the study or on the content of the paper. The authors declare no conflicts of interest.

## Results

275 patients were scheduled to undergo outpatient TKA/THA between December 2015 and June 2017. 261 of those patients had complete data and were included in the analysis: TKA (n = 126) and THA (n = 135). 45 (33%) of THA patients were discharged on the DOS, while 47 (37%) of TKA patients were discharged on the DOS. All patients who were not discharged on the DOS were discharged the following day. Patients in the 2 groups were similar in respect of age, sex, or preoperative OKS/OHS (Tables 1–3).

**Table 2.** Mean Oxford Knee Scores (OKS) in TKA patients

	DDOS	nDDOS	Difference	
OKS	n = 47	n = 79	mean (95% CI)	p-value
Before	22	22	0.2 (–2.0 to 2.4)	0.9
At 3 months	32	31	0.8 (–2.3 to 4.1)	0.6
At 1 year	39	38	1.0 (–1.9 to 3.9)	0.5
Difference				
3 months—before	10	9	0.7 (–2.6 to 4.0)	0.7
1 year—before	16	16	0.8 (–2.2 to 3.9)	0.6

For abbreviations, see Table 1

**Table 3.** Mean Oxford Hip Scores (OHS) in THA patients

	DDOS	nDDOS	Difference	
OHS	n = 45	n = 90	mean (95% CI)	p-value
Before	24	23	0.6 (–1.7 to 2.9)	0.6
At 3 months	39	37	2.2 (–0.5 to 4.8)	0.1
At 1 year	43	43	0.1 (–2.3 to 2.4)	1.0
Difference				
3 months—before	16	14	1.6 (–1.7 to 4.7)	0.4
1 year—before	19	20	0.5 (–4.0 to 2.9)	0.7

For abbreviations, see Table 1

**Table 1.** Demographics and satisfaction. Values are count (%) unless otherwise specified

Factor	DDOS	nDDOS	p-value
Participants, n	92 (	169 (	
Mean age (SD)	60 (11)	62 (11)	0.2
Male	53 (58)	80 (47)	0.2
Female	39 (42)	89 (53)	
THA	45 (33)	90 (67)	0.6
TKA	47 (37)	79 (63)	
Would undergo the same treatment again			
yes	78 (93)	129 (87)	0.05
no	4 (5)	4 (3)	
unsure	2 (2)	15 (10)	
Satisfaction with treatment			
(0–10), median (range)	9 (7–10)	9 (6–10)	0.4

DDOS = discharged on the day of surgery

nDDOS = not discharged on the day of surgery

OKS at 3 months/1year follow-up was 32/39 and 31/38 for DDOS and nDDOS patients, respectively (p = 0.6/p = 0.5). OHS at 3 months/1year follow-up was 39/43 and 37/43 for DDOS and nDDOS patients, respectively (p = 0.1/p = 0.9) (Tables 2 and 3). Furthermore, the 2 groups of patients had similar increases in OHS/OKS at both 3 and 12 months’ follow-up (Tables 2 and 3).

**Figure 1.**
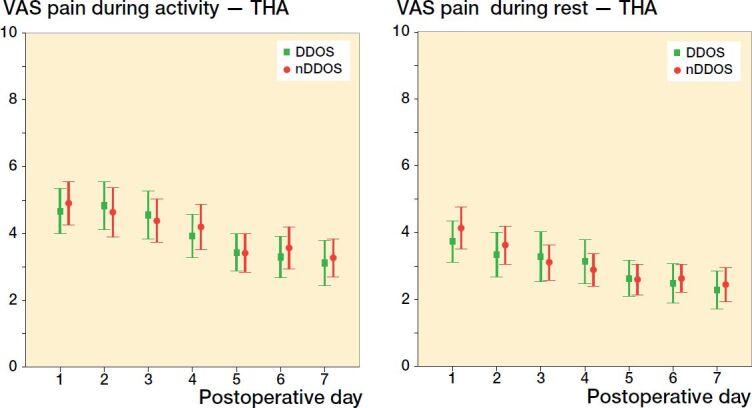
THA VAS scores (mean and 95% CI) for pain during activity (left panel) and during rest (right panel).

**Figure 2.**
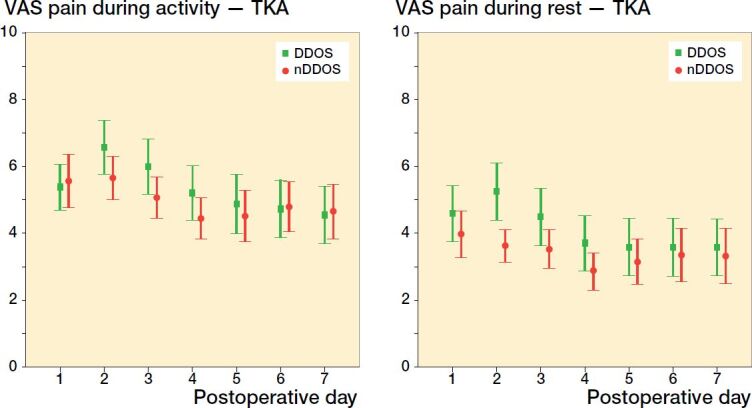
TKA VAS scores (mean and 95% CI) for pain during activity (left panel) and during rest (right panel).

Among THA patients, no statistically significant difference was found regarding pain scores between DDOS and nDDOS patients on POD 1–7 (Figure 1). Among TKA patients, no statistically significant difference was found regarding pain scores with 1 exception: on POD 2 DDOS patients reported a mean VAS score of 5.2 during rest compared with 3.6 among nDDOS patients (p = 0.002) (Figure 2). DDOS TKA patients had a mean VAS score of 4.6 on POD 1 during rest compared with 4.0 among nDDOS TKA patients (p = 0.3). Respectively, these scores dropped to 3.6 and 3.3 on POD 7 during rest (p = 0.7). During activity, DDOS TKA patients scored 5.4 on POD 1 whereas nDDOS TKA patients scored 5.6 (p = 0.7). On POD 7, these scores dropped to 4.6 and 4.7 (p = 0.9), respectively.

**Figure 3.**
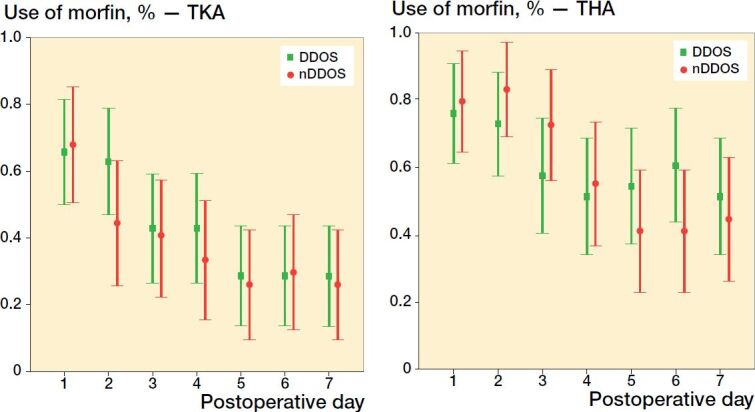
Use of morphine (percentages with 95% CI) among TKA patients (left panel) and THA patient (right panel) .

Regarding use of morphine, a similar pattern was found in both DDOS and nDDOS patients, as there was no statistically significant difference in THA or TKA patients (Figure 3).

Patients from both groups also reported similar satisfaction, as DDOS patients on average rated their experience 9.1/10 compared with nDDOS patients who scored 9.0/10 (p = 0.4) (Table 1). 93% of DDOS patients claimed that they would would go through the process again compared with 87% of nDDOS patients (p = 0.05) (Table 1).

## Discussion

In this prospective cohort study, we found very similar patient-reported outcomes when comparing a group of patients who were discharged on the DOS after THA/TKA with a group of patients who were not discharged on the DOS. This was the case with regards to postoperative OHS and OKS, as well as pain, and use of morphine during the first week after surgery. Furthermore, similar levels of satisfaction were found between the two groups.

Patient-reported outcomes measured using OKS and OHS were similar between the 2 groups prior to surgery and at 3- and 12-months’ follow-up. Change in OKS and OHS at 3- and 12-months’ follow-up were also similar between groups suggesting that the outcome of THA/TKA is not affected negatively by discharge on the DOS. DDOS patients included in this study were able to undergo the same postoperative rehabilitation after discharge as nDDOS patients despite shorter hospital stays and therefore fewer instructions during hospital stay. The mean preoperative OHS in our study was 24 among DDOS patients and 23 among nDDOS patients, which is similar to previous studies investigating early outcome following total joint arthroplasty (Klapwijk et al. 2017, Porsius et al. 2018). Klapwijk et al. (2017) also measured OHS at 6 weeks after THA surgery and found a median OHS of 43 among a mixed group of inpatients and outpatients. The mean preoperative OKS of 22 (DDOS) and 22 (nDDOS) is also similar to a previous study by Hoeffel et al. (2019), who found OKS at 3 months and 1 year after surgery to be mean 35 and 39, respectively. The patients in our study had similar increases in OHS and OKS to the patients in the aforementioned studies, and overall our patients reached expected and satisfying scores.

Pain scores in the first 7 days after surgery, where pain may be most pronounced, did not—with 1 exception on POD 2—differ statistically between groups. However, there seemed to be a trend towards higher pain levels among DDOS TKA patients compared with nDDOS patients on POD 2–4 both at rest and during activity. A possible explanation for this could be that patients discharged on DOS are more active both before and after discharge and therefore have higher pain levels compared with patients who spent 1 night at the hospital. Another potential explanation could be that compliance regarding use of pain medication other than morphine is lower among DDOS TKA patients as they spend a shorter time in hospital and therefore receive more information during a shorter period of time. Generally lower pain scores following THA may explain why no difference is seen between DDOS and nDDOS THA patients. The literature on pain in the first days after surgery is inconsistent as some find differences between inpatients and outpatients with regards to pain (Goyal et al. 2017) whereas others do not (Gauthier-Kwan et al. 2018). Goyal et al. (2017) found that THA outpatients had higher pain levels than inpatients on POD 1 and proposed that insufficient pain management may be the cause of this increased pain among outpatients, which is in line with our findings and proposed explanations—although the difference was among TKA patients and not THA patients in our study. Few studies have compared pain scores between outpatients and inpatients following THA and TKA during the first postoperative days, but other studies have done so with regards to the first postoperative weeks and months. These studies have found similar pain levels between inpatients and outpatients after THA and TKA (Schotanus et al. 2017, Füssenich et al. 2020). Schotanus et al. (2017) found that 6 weeks after TKA both inpatients and outpatients had mean NRS pain scores of 2.6. As expected, those scores are lower than pain scores measured in our study on POD 7 as pain is still expected to decrease after the first week.

Also, the use of rescue medication in the form of opioids was similar between the 2 groups. This goes to show that discharge on the DOS does not lead to increased opioid use among selected patients. Gauthier-Kwan et al. (2018) found similar results, as outpatients and inpatients had statistically similar use of opioids during the first postoperative days. The literature on this subject is limited and further studies are necessary to determine whether or not there is a difference between the 2 groups of patients.

Patients having THA or TKA are generally satisfied with their overall experiences (Husted et al. 2008, Hoeffel et al. 2019). That was also the case in this study as the 2 groups of patients on average scored 9.0/10 and 9.1/10, respectively. These findings are in accordance with an earlier study (Kelly et al. 2018).

Furthermore, the willingness to do the same procedure under the same circumstances again was similar in both groups. A nearly statistically significant difference in willingness to undergo the same procedure was found between the 2 groups of patients, with slightly more DDOS patients responding that they were willing to undergo the same procedure again. Porsius et al. (2018) found similar levels of willingness to undergo fast-track THA among three different groups of patients: fast-, average-, and slow-recovery patients. A large proportion of the fast-recovery patients had outpatient surgery compared with the proportion of slow-recovery patients, which goes to show that outpatients are not less satisfied than inpatients after THA.

Though this study sheds light on some aspects of patient-reported outcomes and pain after THA and TKA, there are also limitations associated with this study. 1 of the limitations of our study lies in possible differences between the 2 groups of patients as this was a prospective cohort study without randomization. This allows for potential confounding between the 2 groups. Even though recorded patient demographics and preoperative PROMS were similar between groups, other non-accounted for factors may be responsible for some patients being discharged on the DOS, while others were not. As this study is observational, there is a potential for both selection bias and residual confounding. However, we chose not to adjust the analysis for potential bias but present the PROM data for the 2 cohorts as is, to present the actual clinical reality for such patients scheduled for outpatient surgery. Patients’ mentality may play a role with some patients having a more positive attitude toward DOS discharge. Such patients might be more likely to report high levels of satisfaction, provided they were discharged on the DOS; this, however, is speculative. Furthermore, this study would be strengthened by additional data, as the reasons for inpatients’ overnight stays were not registered. The differences in the use of physiotherapy between the 2 groups of patients following discharge may be a confounding variable in this study, though it seems unlikely. Tracking the use of physiotherapy is not possible as it takes place outside of the hospital after discharge. Additionally, a potential bias could exist in the form of a difference in pain between the two groups of patients. One could argue that increased pain after surgery could lead to a higher probability of overnight stay in hospital but this study did not aim to investigate reasons for overnight stay in hospital. Finally, external validity is a limitation, as patients scheduled for outpatient surgery in this study may differ compared with other setups, making results more difficult to apply to a different patient population.

In conclusion, in this prospective cohort study, we found similar pain scores, use of rescue medication (morphine), and OHS/OKS up to 1 year reported by THA and TKA patients discharged on the day of surgery and patients who stayed at least 1 night in the hospital. These findings support continuous utilization of outpatient TKA and THA in selected patients.
